# Replication-Coupled Recruitment of Viral and Cellular Factors to Herpes Simplex Virus Type 1 Replication Forks for the Maintenance and Expression of Viral Genomes

**DOI:** 10.1371/journal.ppat.1006166

**Published:** 2017-01-17

**Authors:** Jill A. Dembowski, Sarah E. Dremel, Neal A. DeLuca

**Affiliations:** Department of Microbiology and Molecular Genetics, University of Pittsburgh School of Medicine, Pittsburgh, Pennsylvania, United States of America; Emory University, UNITED STATES

## Abstract

Herpes simplex virus type 1 (HSV-1) infects over half the human population. Much of the infectious cycle occurs in the nucleus of cells where the virus has evolved mechanisms to manipulate host processes for the production of virus. The genome of HSV-1 is coordinately expressed, maintained, and replicated such that progeny virions are produced within 4–6 hours post infection. In this study, we selectively purify HSV-1 replication forks and associated proteins from virus-infected cells and identify select viral and cellular replication, repair, and transcription factors that associate with viral replication forks. Pulse chase analyses and imaging studies reveal temporal and spatial dynamics between viral replication forks and associated proteins and demonstrate that several DNA repair complexes and key transcription factors are recruited to or near replication forks. Consistent with these observations we show that the initiation of viral DNA replication is sufficient to license late gene transcription. These data provide insight into mechanisms that couple HSV-1 DNA replication with transcription and repair for the coordinated expression and maintenance of the viral genome.

## Author Summary

HSV-1 is a ubiquitous human pathogen that causes persistent infections for the lifetime of the infected host. Of major interest are the mechanisms underlying how the virus utilizes cellular resources to rapidly replicate with high fidelity. We show that DNA repair and late transcription are coupled to genome replication by identifying the viral and cellular factors that associate with replicating viral DNA. In addition to transcription and repair, the results also describe how RNA processing and virion packaging are temporally coordinated relative to genome replication.

## Introduction

Herpesviruses belong to a large family of enveloped double-stranded DNA viruses that cause persistent infections in a broad range of metazoan hosts. Much of the infectious cycle occurs in the nucleus of cells, including events underlying transitions between latent and productive states, viral gene expression, DNA replication, and packaging of nascent genomes into capsids. Herpesviruses share strong evolutionary relationships with their hosts and have evolved mechanisms to subvert host antiviral restriction pathways and DNA damage responses, while adapting host mechanisms for viral genome maintenance and gene expression [1,2]. Although virus-host interactions largely determine the outcome of infection, there is limited understanding of how the dynamic interplay between host factors and viral DNA contribute to processes that occur on viral genomes.

Herpes simplex virus type 1 (HSV-1) is a ubiquitous human pathogen that causes recurrent contagious oral and genital sores and serious infections of the eye and central nervous system. HSV-1 consists of a 152 kilobasepair linear genome that codes for over 80 proteins [3,4]. Like all herpesviruses, during productive infection, HSV-1 largely utilizes its own DNA synthetic machinery for genome replication but depends on host RNA polymerase II (Pol II) for the transcription of viral genes [5].

HSV-1 DNA replication requires at least seven viral DNA replication proteins [6] and initiates at least one of three origins of replication [7]. These sites are bound by the origin binding protein (UL9) [8], which in combination with the single-stranded DNA binding protein (ICP8) distorts the origins [9]. This is followed by unwinding and priming by the helicase/primase complex (UL5/UL8/UL52) for the activation of DNA synthesis by the two subunit viral DNA polymerase (catalytic subunit UL30, processivity subunit UL42) [10]. While this set of viral proteins includes a DNA-dependent DNA polymerase and functional analogs of other cellular replication proteins, it is not sufficient to drive origin-dependent replication in vitro. This implies the requirement for additional viral or cellular proteins. Furthermore, the existence of branched, concatemeric, and isomeric viral DNA structures suggests that alternative modes of replication involving recombination must also exist [11–15].

Although the viral DNA polymerase has a high probability to incorporate incorrect bases, the HSV-1 genome is maintained with high fidelity [16]. Furthermore, the genome is known to contain several nicks and gaps [17–19], which would likely impede replication and transcription. Some cellular DNA repair proteins have been implicated in productive stages of infection including those involved in mismatch repair (MMR) and double strand break (DSB) repair [1]. However, HSV-1 inhibits classic cellular DSB repair pathways including nonhomologous end joining and homologous recombination. It is therefore likely that the virus adapts cellular repair proteins to mediate processes for virus-specific genome maintenance.

HSV-1 transcription occurs through a tightly ordered cascade of immediate early (IE)(α), early (β), and late genes (γ) [20,21]. IE genes are expressed without de novo viral protein synthesis and include regulatory factors that alter host cell metabolism and control viral gene expression. The IE gene product ICP4 is a transcriptional repressor of IE genes and activator of early and late genes [22–24]. Early gene products include viral DNA replication factors and late gene products include virion assembly and structural proteins. Late genes are further classified into leaky late or true late, depending on the extent to which they depend on viral DNA replication for expression. True late gene expression strictly depends on viral DNA synthesis, although mechanisms by which transcription and replication are coupled are not understood.

Although multiple aspects of the HSV-1 infectious cycle have been carefully investigated, the role viral and cellular factors play in nuclear events that coordinate viral DNA replication with recombination, repair, and late gene expression are not well understood. We recently adapted the iPOND (isolation of proteins on nascent DNA) method [25] to purify viral genomes from infected cells for the comprehensive analysis of viral genome associated proteins by mass spectrometry [26]. This study provided insight into cellular factors and processes that act on viral genomes at various times during infection. In the current study, we describe the purification and imaging of HSV-1 replication forks for the elucidation of proteins functioning on replicating genomes. Viral replication factors were identified at sites of active DNA synthesis, as well as groups of proteins that have known functions in specific cellular processes, including DNA repair and transcriptional regulation. Pulse chase and imaging studies reveal the temporal and spatial relationships of these proteins with viral DNA with respect to replication and provide insight into mechanisms that coordinate the repair and expression of viral genomes with genome replication. To our knowledge this is the most comprehensive analysis of cellular factors associated with viral replication forks to date.

## Results

### Kinetics of HSV-1 DNA Replication and Resolution of Replication Fork Pulse Chase Assays

**Fig 1:**
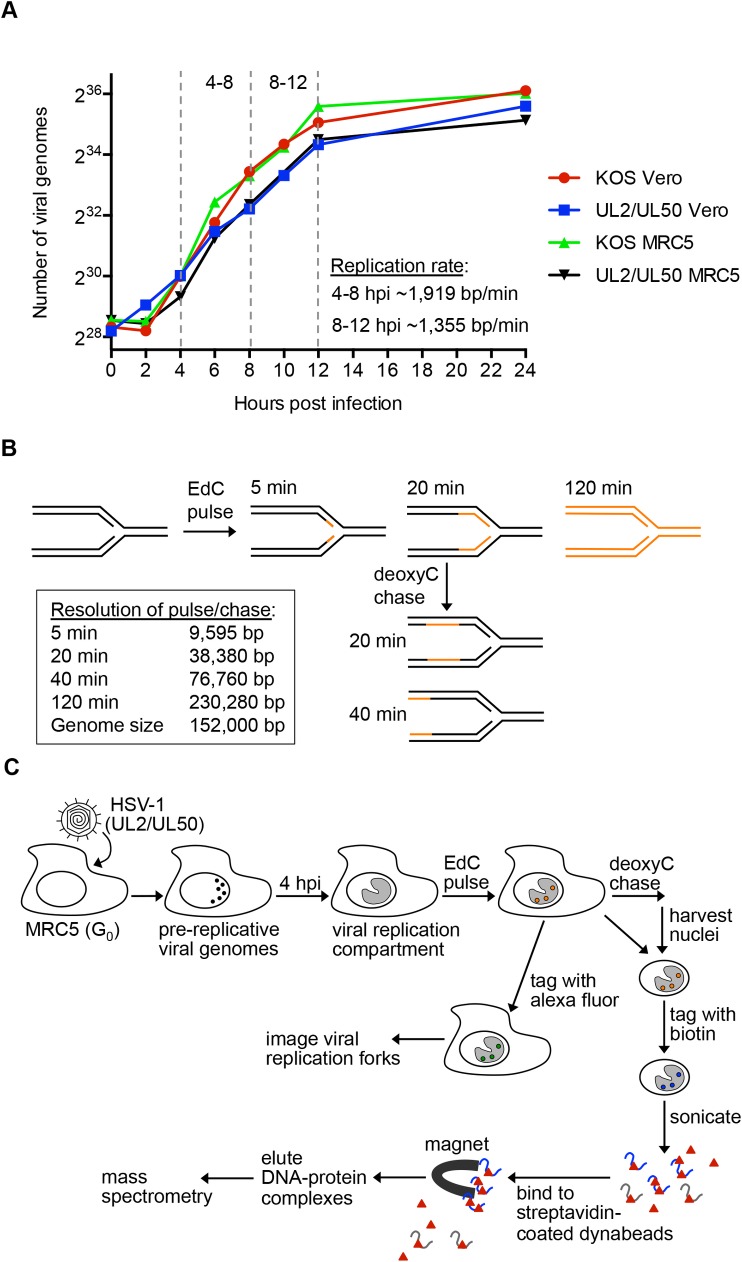
Methodology to Label, Image, and Purify HSV-1 Replication Forks. A. Calculation of the rate of viral DNA replication. MRC5 or Vero cells were infected with KOS or UL2/UL50 and the number of viral genomes as a function of hours post infection (hpi) was determined by quantitative PCR (qPCR). The shown replication rates at 4–8 and 8–12 hpi were calculated using UL2/UL50 infected MRC5 cells because these conditions were used for subsequent proteomic analyses. B. Calculated resolution of pulse/chase experiments. Number of base pairs labeled or chased for various time intervals are indicated. C. Schematic of methods used to label and tag viral replication forks. Pre-replicative genomes coalesce and form replication compartments by 4 hpi. After pulse chase of viral replication forks, DNA was either tagged with an alexa fluor for imaging or biotin for purification following isolation of nuclei. For isolation of fork associated proteins, nuclei were lysed and DNA was fragmented by sonication and DNA-protein complexes were purified on streptavidin-coated beads. DNA protein complexes were eluted from beads after several wash steps (not depicted) and proteins were identified by mass spectrometry.

We previously established methods based on iPOND to label HSV-1 DNA with clickable nucleotide analogs (EdU or EdC) for the covalent attachment of biotin or a fluorophore for purification or imaging of viral genomes [26]. We sought to adapt these methods to selectively label, purify, and image viral replication forks to investigate replication-coupled events. In order to examine the timing of protein interactions with viral replication forks, we first determined the rates of viral DNA replication throughout productive infection (Fig 1A). Infections, quantification of viral DNA, and calculation of replication rates were carried out as described in the Materials and Methods. The rates of HSV-1 DNA replication, calculated as the number of base pairs (bp) synthesized per minute (min), consistently fluctuate from 4–8, 8–12, and 12–24 hours post infection (hpi) (dashed vertical lines), with replication occurring more rapidly earlier during infection. The rates of DNA synthesis range from 1384–2184 bp/min from 4–8 hpi and 1020–1448 bp/min from 8–12 hpi. These rates may represent the use of multiple origins of replication on a single viral genome and therefore DNA synthesis by an individual polymerase is likely slower than the calculated rate.

To determine the resolution of pulse chase assays for the purification of viral replication fork associated proteins at 4 hpi, the number of base pairs labeled during the time intervals used in subsequent experiments were calculated (Fig 1B). These values do not take into account the amount of time it takes for EdC to be incorporated into cells and phosphorylated. Therefore, these values represent the maximum number of base pairs labeled and chased by a single polymerase. A 5–20 min pulse with EdC should provide accurate representation of viral replication forks and a 120 min pulse would result in labeling of entire viral genomes.

### Viral Replication Proteins and Cellular DNA Repair and Transcription Factors are Enriched on Viral Replication Forks

To identify factors and complexes enriched on HSV-1 replication forks, we pulse labeled viral DNA with EdC for 5 or 20 min, tagged and isolated labeled DNA, and identified associated proteins by mass spectrometry (Fig 1C). MRC5 cells were used in this study because they enter a quiescent state in which cellular DNA replication is arrested once grown to confluency ensuring that only viral DNA is labeled and purified in our assays (S1 Fig) [26]. Cells were infected with the virus UL2/UL50, which is defective for the viral uracil glycosylase (UL2) and dUTPase (UL50), resulting in greater incorporation of EdU and EdC into viral DNA [26]. For maximum protein yield, we adapted the accelerated native iPOND (aniPOND) method [27], which involves purification of DNA-protein complexes under native conditions and results in higher protein yield compared to the standard iPOND method. We improved this method by switching to magnetic streptavidin-coated beads for purifications, which resulted in reproducibly higher protein yield and decreased background binding.

**Fig 2:**
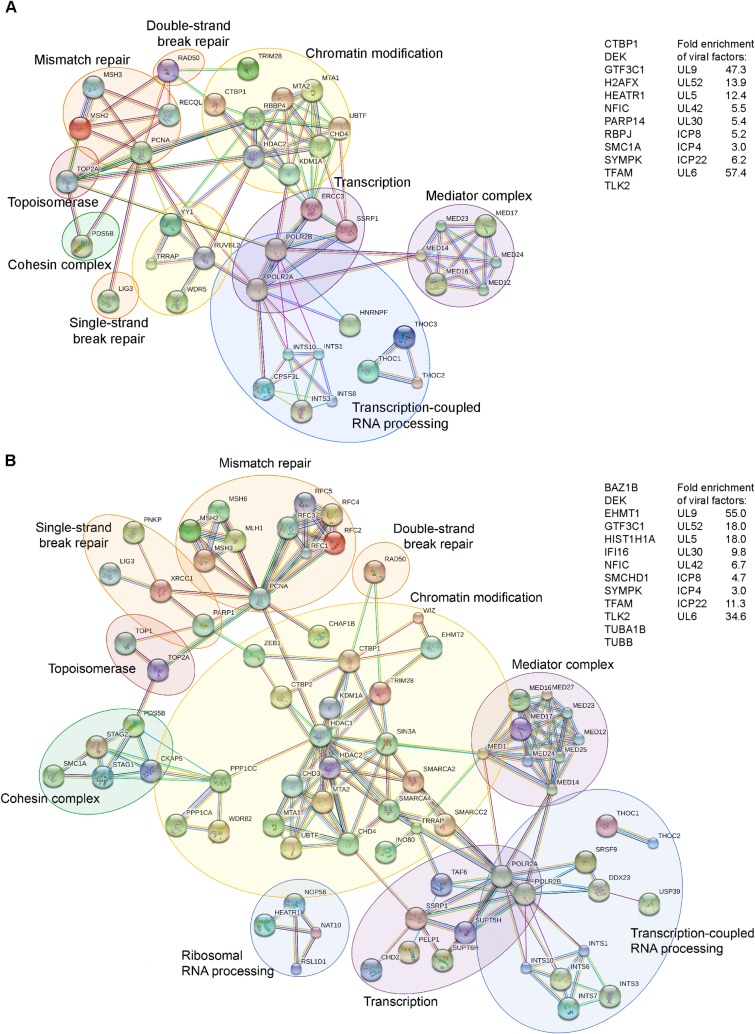
Viral Replication Proteins and Select Cellular Factors are Associated with HSV-1 Replication Forks. A. STRING mapping of proteins enriched on viral replication forks after a 5 min EdC pulse. Human proteins enriched by 5 fold compared to the unlabeled negative control are shown in the functional interaction map or list of unmapped proteins, which was generated using STRING [28] with data settings to display only high confidence interactions. Gene names were used to map interactions. Circles indicate proteins that function in the same biological process. Fold enrichment of viral proteins is shown at right. B. STRING mapping of proteins enriched by 8 fold on viral replication forks after a 20 min pulse. See also S1 Table.

Proteins enriched relative to the unlabeled control after a 5 min pulse with EdC were mapped using the STRING functional protein interaction database [28] (Fig 2A). A total of 52 human proteins with known functions in DNA repair, chromatin modification, transcription, and transcription-coupled processes were identified as associated with replication forks. Output from the STRING database indicated that there were more interactions among the identified proteins than would be expected for a random set of proteins. In addition, the viral replication proteins UL9, UL52, UL5, UL42, UL30, and ICP8 were enriched on pulse labeled DNA by at least 5 fold. The helicase-primase complex associated protein UL8 was not identified, perhaps because it is more transiently associated with viral DNA. Importantly, cellular DNA polymerases were not identified, consistent with the selective purification of viral and not cellular replication forks in our assays.

Proteins enriched after a 20 min pulse are shown in Fig 2B. A total of 90 human proteins enriched by at least 8 fold compared to the unlabeled negative control were mapped using STRING, revealing striking similarities between the 5 and 20 min pulse datasets. In fact, most human proteins enriched on replication forks pulse labeled for 5 min were found after 20 min. More members of the same complexes were identified in the 20 min pulse compared to the 5 min pulse. These data implicate specific repair processes at replication forks including MMR and single strand break (SSB) and DSB repair, as well as the chromatin remodeling complexes INO80, NuRD, and FACT. Core complexes involved in Pol II mediated transcription were also present including Mediator, Integrator, TFIID, and Pol II. These data provide evidence for the coupling of repair, chromatin remodeling, and transcription to viral DNA replication.

### Analysis of Protein Dynamics with Respect to HSV-1 Replication Forks

**Fig 3:**
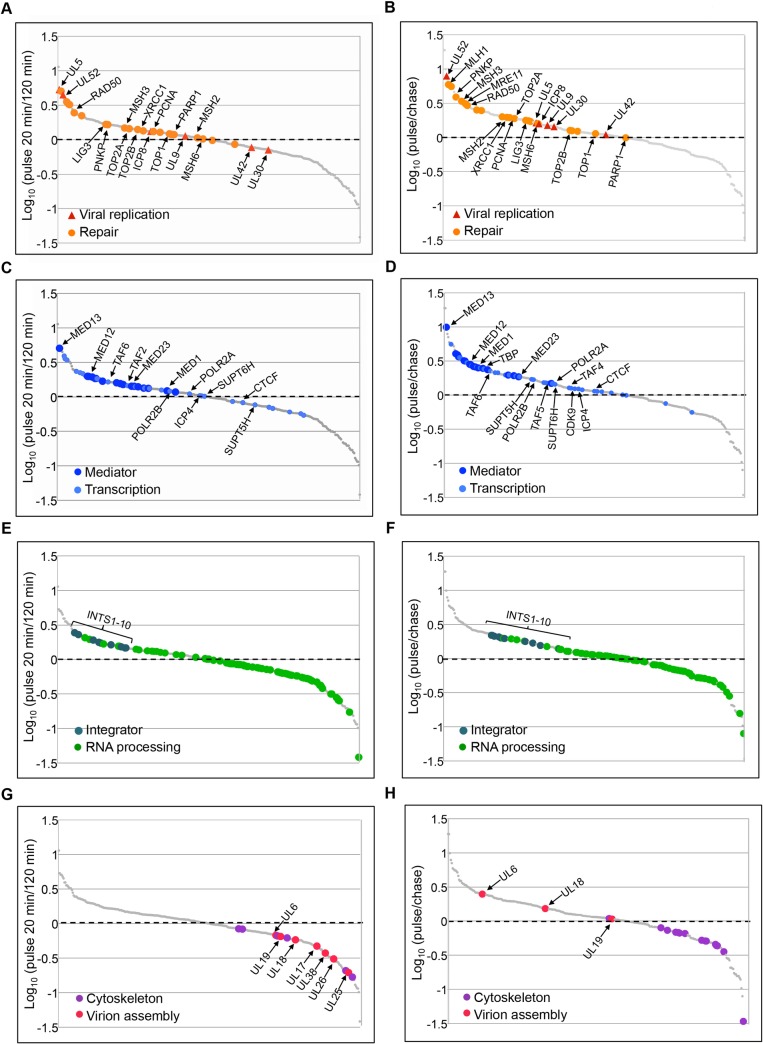
Pulse and Pulse Chase Analysis of Viral Replication Forks Reveals Interaction Dynamics Between Nascent Viral DNA and Associated Proteins. Relative fork associated protein levels were compared between pulse labeled replication forks (20 min) and replicated genomes (120 min) (A, C, E, G) and between pulse labeled (pulse) and chased (chase) replication forks (B, D, F, H). Viral replication and cellular repair proteins (A, B), transcription factors (C, D), RNA processing factors (E, F), and cytoskeletal and virion assembly proteins (G, H) are highlighted in individual graphs. Gene names are indicated for corresponding proteins. See also S2 Fig and S1 Table.

To compare the relative abundance of proteins on viral replication forks to whole viral genomes, infected cells were pulse labeled with EdC for 20 min to label replication forks, or for 120 min to label entire viral genomes (Fig 1B) followed by the isolation of DNA for the identification of associated proteins by mass spectrometry (C). Spectral count (SpC) values for identified proteins were normalized to the total abundance of proteins in that sample as described in the Materials and Methods to account for differences in the amount of total DNA isolated in each condition. Ratios of the normalized values (pulse 20 min/120 min) were compared for individual proteins, which were then graphed to highlight trends associated with factors that function in the same biological processes (Fig 3A, 3C, 3E and 3G and S2A Fig). Factors that were greatly enriched near replication forks include the HSV-1 replication helicase (UL5) and primase (UL52), the DSB repair protein RAD50, the MED12 and MED13 subunits of the Mediator complex, Integrator complex members, and the INO80 complex. Proteins that were more enriched on whole genomes after a 120 min pulse include factors involved in transcription-coupled RNA processing and virion assembly, as well as components of the cellular cytoskeleton and select histones.

To further investigate the dynamics of protein interactions with respect to viral replication forks, pulse chase analysis was carried out. Infected cells were pulse labeled with EdC for 20 min to label replication forks or pulsed for 20 min and then chased for 40 min with deoxyC (Fig 1B) followed by the isolation of DNA and identification of associated proteins (C). Ratios of the normalized SpC values (pulse/chase) were graphed for individual proteins (Fig 3B, 3D, 3F and 3H and S2B Fig). Factors that were greatly enriched in the pulse compared to chase include UL52, several DNA repair proteins, MED12 and MED13, the Integrator complex, the NuRD complex, and the viral portal protein UL6. Proteins that were more enriched in the chase include RNA processing factors and cytoskeletal proteins.

Western blot analysis was used to compare the interaction dynamics of the viral replication proteins ICP8 and UL42 with respect to replication forks (S2C Fig). ICP8 was enriched in the pulse and not in chases and relative levels of UL42 did not change significantly under these conditions. ICP8 binds to single stranded DNA near replication forks and UL42 binds to double stranded DNA nonspecifically. These data suggest that UL42 remains associated with DNA after it is deposited at sites of viral replication and validate trends observed for these two proteins by mass spectrometry (Fig 3B).

### Sites of Active Viral DNA Synthesis are Localized to Distinct Foci within HSV-1 Replication Compartments

**Fig 4:**
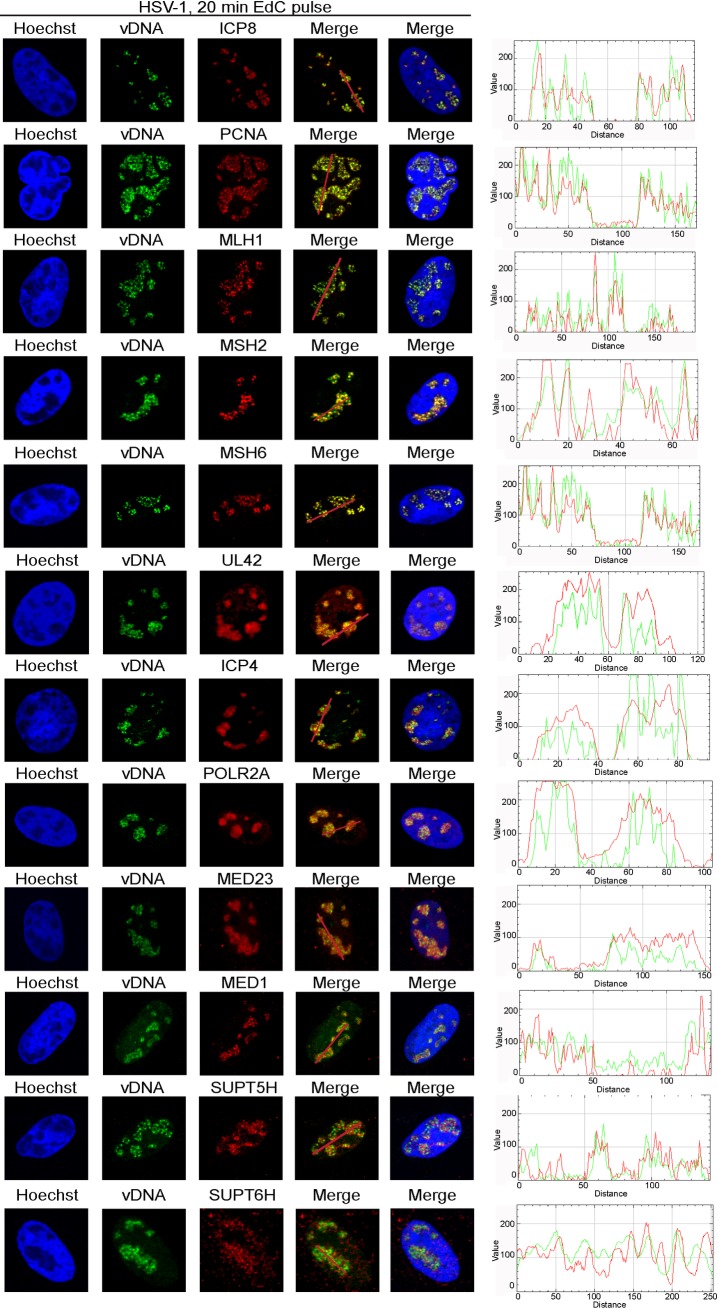
Proteins Enriched on Viral Replication Forks Colocalize with Sites of Active Viral DNA Synthesis within HSV-1 Replication Compartments. Sites of active viral DNA synthesis were labeled with EdC for 20 min after a four-hour infection of Vero cells with wild type HSV-1. Infected cells were fixed and EdC labeled DNA was tagged with alexa fluor 488 to visualize viral replication forks (green) and viral and cellular proteins were visualized by immunofluorescence (red). Nuclei were labeled with Hoechst (blue). Traces were generated using the RGB profiler plugin in Image J and correspond to the red line drawn on the red/green merge panel. Gene names that correspond to detected proteins are indicated for consistency with proteomics data. See also Table 1.

After the onset of viral DNA replication many nuclear processes in the viral life cycle occur in viral replication compartments [29]. To visualize viral replication forks relative to replication compartments, Vero cells were infected with HSV-1 strain KOS for four hours and actively replicating viral DNA was pulse labeled with EdC for 20 min followed by fixation, click chemistry to tag labeled DNA, and immunofluorescence for ICP8 (Fig 4, top panel). Distinct foci containing labeled viral DNA were visualized within viral replication compartments within the nuclei of infected cells. These foci colocalized with ICP8 (see red/green trace at right), and could be chased away from ICP8 containing foci (S2D Fig). These data demonstrate that distinct sites of viral DNA replication exist within viral replication compartments and provide a tool to visualize and validate the associations of proteins identified by mass spectrometry with sites of active viral DNA synthesis.

### MMR Proteins and PCNA Selectively Associate with HSV-1 Replication Forks and Depend on DNA Replication for Colocalization with Viral DNA

Cellular MMR proteins and PCNA were among the proteins identified to be enriched on viral replication forks after a 5 or 20 min pulse (Fig 2) and were relatively more abundant on pulse labeled compared to chased DNA (Fig 3B). We therefore predicted that these factors should colocalize with sites of active viral DNA synthesis within the nuclei of infected cells. We carried out imaging of viral replication forks as described above coupled with immunofluorescence of MMR proteins (MLH1, MSH2, and MSH6) and PCNA and demonstrated that these proteins do in fact colocalize with sites of active viral DNA synthesis (Fig 4). Compared to mock infected cells (S3 Fig), these factors undergo robust redistribution to viral replication forks within infected cells. In contrast, UL42 localizes to sites of active DNA synthesis, as well as to adjacent sites within replication compartments, consistent with observations from pulse chase mass spectrometry (Fig 3B) and western blot studies (S2C Fig). Taken together, imaging studies corroborate results from viral replication fork pull down assays.

To better understand the relationships of MMR proteins and PCNA with viral DNA, we investigated the recruitment of these factors to viral DNA early during infection before the onset of replication (S3 Fig and Table 1) and to viral genomes inhibited for replication by the addition of the viral DNA polymerase inhibitor acyclovir (S4 Fig and Table 1). In all cases, these factors depend on ongoing viral DNA synthesis to associate with HSV-1 DNA. It can therefore be concluded that PCNA and MMR proteins are recruited to and likely act at viral replication forks during productive HSV-1 infection.

**Table 1:** Proteins that Colocalize with HSV-1 Replication Forks.

Protein	Replication Forks	Input 2 hpi	ACV 6 hpi
ICP8	Yes	Yes (when expressed)	Yes
PCNA	Yes	No	No
MLH1	Yes	No	No
MSH2	Yes	No	No
MSH6	Yes	No	No
UL42	Yes	No^a^	Sometimes
ICP4	Yes^b^	Yes	Yes
POLR2A	Yes^b^	Yes	Yes
MED23	Yes^b^	Yes	Yes
MED1	Yes^b^	Yes	Yes
SUPT5H	Yes^c^	Yes	Sometimes
SUPT6H	Yes^c^	Not tested	Not tested

Viral replication fork associated proteins (listed by gene name) were analyzed for colocalization with pre-replicative EdC-labeled HSV-1 genomes at 2 hpi (Input 2 hpi) and EdC-labeled viral genomes that were inhibited for DNA synthesis by the addition of 100 μM acyclovir throughout infection and imaged at 6 hpi (ACV 6 hpi).

^a^UL42 is not abundantly expressed at 2 hpi [31].

^b^Proteins that colocalize with viral replication forks but not exclusively.

^c^Proteins that appear to localize adjacent to replication forks. See also Fig 4, S3 Fig and S4 Fig.

### Cellular Transcription Factors Colocalize with Sites of Active Viral DNA Replication

Cellular Pol II and the Mediator complex were among proteins enriched on viral replication forks after a 5 or 20 min pulse (Fig 2) and transcription elongation factors SUPT5H (Spt5) and SUPT6H (Spt6) were abundant after a 20 min pulse. These factors were also relatively more abundant on pulse labeled DNA compared to chased DNA (Fig 3D), although the relative level of enrichment varies greatly between these proteins. We therefore predicted that these factors should colocalize with sites of active viral DNA synthesis. We carried out imaging of viral replication forks coupled with immunofluorescence and demonstrated that Pol II (POLR2A), MED23, MED1, SUPT5H, and SUPT6H do in fact colocalize with sites of active viral DNA synthesis, however, not exclusively (Fig 4). POLR2A, MED23, and MED1 also associate with adjacent sites within replication compartments that are not in the act of viral DNA synthesis and SUPT5H and SUPT6H appear to localize adjacent to sites of active DNA synthesis. POLR2A and MED23 have similar distributions within replication compartments as ICP4, consistent with more general roles in the regulation of replication-dependent transcription of late viral genes, as well as replication-independent transcription of IE and early viral genes. Compared to mock infected cells (S3 Fig), these factors were redistributed to viral replication compartments during infection, consistent with the overall reduction in Pol II occupancy on cellular promoters [30]. We observed recruitment of transcription factors to viral genomes early during infection before the onset of replication (S3 Fig and Table 1) and to viral genomes inhibited by acyclovir (S4 Fig and Table 1). Taken together, these data are consistent with the involvement of cellular transcription factors in replication-dependent and replication-independent transcription of viral genes.

### Initial Rounds of Replication are Sufficient to Drive Robust Late Gene Expression

**Fig 5:**
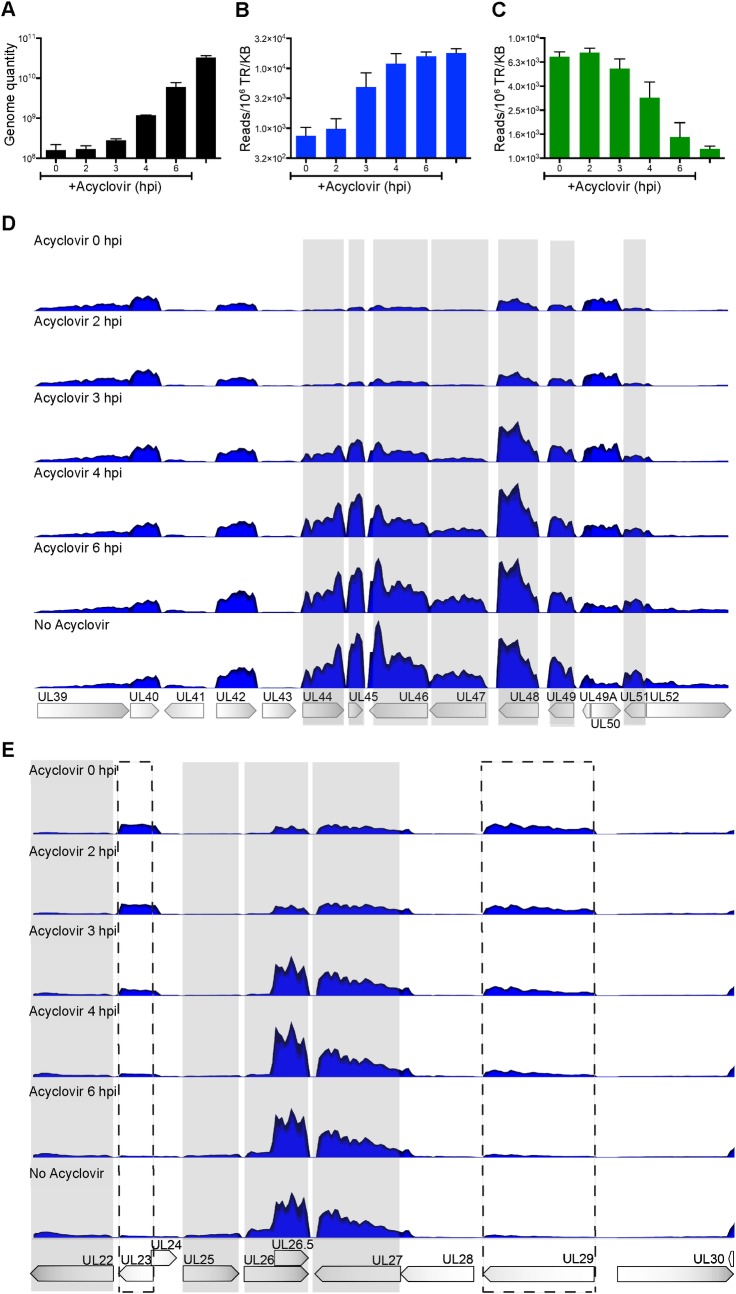
Initial Rounds of Replication are Sufficient for Late Gene Expression. Vero cells were infected with wild type HSV-1 at an MOI of 10 PFU/cell and acyclovir was added to the growth medium as indicated at 0, 2, 3, 4, or 6 hpi. Total DNA or RNA was collected at 12 hpi. A. The number of viral genomes was measured by quantification of the gC gene by qPCR. Error bars represent the standard deviation of biological duplicates. B-E. The quantities of viral transcripts were measured using RNA-Seq. B. RNA-Seq analysis of the late gene UL44 (gC). The units on the y-axis are reads mapped to the viral gene locus per million total reads per kilobase pair (Reads/10^6^ TR/KB). C. RNA-Seq analysis of the early gene UL23 (tk). D. Mapped reads are shown relative to the HSV-1 gene loci from UL39 to UL52. Each sample was normalized relative to 10^7^ total reads. The y-axis maximum value is 75,000 reads. The gray shaded areas indicate late genes. E. Mapped reads are shown relative to the KOS gene loci map from UL22 to UL30. Dashed boxes indicate early genes UL23 (tk) and UL29 (ICP8).

To investigate the coupling of viral transcription to replication, we carried out RNA-Seq to quantify the dependence of viral gene expression on DNA replication. Acyclovir was added to cultures of KOS infected Vero cells at 0, 2, 3, 4, or 6 hpi and DNA or RNA was harvested at 12 hpi to compare the relative abundance of viral genomes and transcripts as a function of time of inhibition of viral DNA synthesis. The control was harvested at 12 hpi without the addition of acyclovir. Acyclovir inhibits viral DNA replication by ~27 fold when added at 4 hpi compared to the control (Fig 5A), but only inhibits transcription of the true late gene UL44 (glycoprotein C, gC) by 1.5 fold when added at this time (B). The addition of acyclovir at 4 hpi also resulted in a 2.5 fold increase in expression of the early gene UL23 (thymidine kinase, tk) compared to the control (C), whose expression typically peaks between 3 and 4 hpi [31]. Additional late genes follow the same kinetics for activation as gC (D, E, shaded) and the early gene UL29 (ICP8) follows the same kinetics as UL23 (E, dashed box). Taken together, these data indicate that initial rounds of viral DNA replication are sufficient to alter the transcriptional landscape of HSV-1 genomes enough to activate late gene and alter early gene expression.

## Discussion

The processes of genome replication, transcription, repair, and maintenance are often coupled in various ways in cells to allow for growth, differentiation, and development. The coupling of these processes is also fundamental to the life cycle of complex DNA viruses such as HSV-1. The expression of approximately 80 viral genes is coordinated with the entry of the viral genome into the nucleus and replication of viral DNA. In addition, the genome undergoes prolific recombination, however it remains relatively stable from a genetic standpoint. These processes, along with genome maturation and packaging, are coordinated by the interactions of viral and cellular factors with the viral genome such that the first progeny virus is assembled within the first 4–6 hours post infection. A pivotal coordinating event is viral DNA replication.

**Fig 6:**
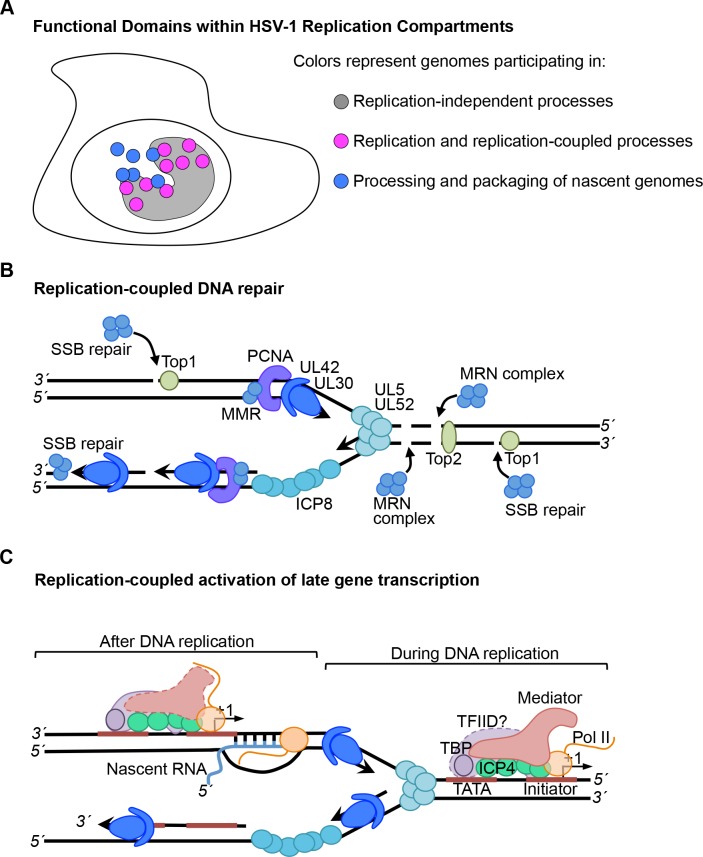
Model Illustrating HSV-1 Replication-Coupled Processes. A. Subdomains within HSV-1 replication compartments contain genomes participating in replication-coupled and replication-independent processes. B. Model depicting replication-coupled DNA repair and C. transcription.

Here we developed methods to selectively purify and image HSV-1 replication forks for the temporal and spatial analysis of protein interactions with sites of viral DNA synthesis to ascertain how these fundamental processes are coupled. We demonstrate that select cellular factors copurify with nascent viral DNA, which include factors that function in SSB and DSB repair, MMR, transcription, and chromatin remodeling (Fig 2). We further demonstrate that identified factors colocalize with distinct sites of ongoing DNA synthesis within replication compartments (Fig 4), confirming the coupling of repair and transcription to viral DNA replication. We test for the dependence of these interactions on initial rounds of viral DNA synthesis and demonstrate that recruitment of PCNA and MMR proteins to viral DNA is strictly replication-dependent. On the other hand, Pol II and Mediator interactions are both replication-coupled and independent (S3 Fig and S4 Fig), consistent with replication-dependent and independent modes of gene expression. Furthermore, we track nascent viral DNA out of replication compartments (S2D Fig), where the newly replicated DNA makes more contact with factors involved in transport and packaging of viral genomes (Fig 3G and 3H) or transcription-coupled RNA processing (E, F). The ability to recognize and track distinct populations of viral DNA with respect to DNA synthesis reveals the existence of functional subdomains within HSV-1 replication compartments (Fig 6A).

### Dynamic Associations of Viral Factors with Nascent Viral DNA

We demonstrate that at 4 hpi viral replication proteins are abundantly associated with sites of viral DNA synthesis (Fig 2). After longer pulses with EdC (Fig 3A) or in pulse chase experiments (B), the levels of the origin binding protein UL9 do not change significantly, implicating that UL9 binds to origins during initial rounds of replication and that the relative levels on individual genomes remains constant from 4–6 hpi. Pulse chase studies reveal close associations between UL5/UL52 and sites of active viral DNA synthesis (Fig 3A and 3B). Levels decrease significantly in chases, consistent with close associations with replication forks and indicating that these proteins are not deposited onto newly replicated DNA. In contrast, UL42 remains associated in chases (Fig 3B and S2C Fig) and does not exclusively colocalize with replication foci within replication compartments (Fig 4). It is possible that UL42 remains associated with nascent viral DNA through sequence nonspecific interactions with double-stranded DNA. This is in stark contrast to the pulse chase and binding properties of ICP8 (S2C Fig and Fig 3B), which selectively associates with single stranded DNA at replication forks (Fig 4).

Viral transcription factors ICP4 and ICP22 also associate with sites of viral DNA synthesis (Fig 2), consistent with functions in replication-dependent late gene expression. Levels of these factors remain unchanged in pulse experiments (Fig 3C) implicating ongoing functions in viral gene expression from 4–6 hpi. Furthermore, levels remain unchanged in pulse chase experiments (D), consistent with these proteins being deposited on and remaining associated with newly replicated DNA. ICP4 associates with sites of active viral DNA synthesis, as well as with adjacent sites within replication compartments (Fig 4), consistent with the ability to associate with double stranded DNA in a sequence nonspecific manner [32], although site specific binding across the genome also occurs [33,34].

An interesting observation from these studies is that the viral portal protein UL6 is abundant on viral replication forks (Fig 2). In fact, it is one of the most abundant proteins associated with pulse labeled viral DNA. This protein is also more closely associated with sites of active viral DNA synthesis because the relative levels of UL6 decrease as you move away from replication forks (Fig 3H). This is in contrast to other viral DNA processing and packaging factors, which are only enriched on viral genomes after longer pulses with EdC (G). It is possible that UL6 plays a yet to be identified role in viral DNA replication in addition to its function as the portal for entry into the HSV-1 capsid.

### Cellular Repair Proteins Interact with Viral Replication Forks

Among proteins enriched on viral replication forks are factors that function in the repair of damaged cellular DNA (Fig 2). These include cellular proteins with known roles in MMR, SSB repair, and DSB repair. In general these proteins may function in the repair of damaged DNA during replication or may mediate DNA recombination.

### Mismatch Repair

The MMR proteins, MSH2, MSH3, and MSH6 are among the proteins most abundantly enriched on viral replication forks. After a 20 min pulse, MSH2 and MSH6 are enriched on EdC labeled DNA by ~100 fold and MSH3 by 30 fold compared to the unlabeled negative control (Fig 2). MLH1 was less abundant but still enriched by ~9 fold. The MSH2-MSH6 heterodimer accounts for 80–90% of cellular MSH2 and preferentially recognizes base pair mismatches and small insertion/deletion mutations [35]. These proteins have previously been shown to copurify with viral genomes [26] and with ICP8 from virus infected cells [36]. MSH2, MSH6, and MLH1 have also been shown to colocalize with viral replication compartments [36,37] and knockdown of MSH2, MSH6, and MLH1 results in reduced viral yields and defects associated with replication compartment formation [37]. Here we demonstrate that these proteins colocalize with sites of active viral DNA synthesis within replication compartments (Fig 4), depend on DNA replication for colocalization with viral DNA (S3 Fig and S4 Fig), and are more closely associated with pulse labeled replication forks rather than chased DNA (Fig 3B). Our studies place the cellular MMR machinery at or near viral replication forks and demonstrates an intimate relationship between viral DNA replication and MMR. Because UL30 incorporates incorrect nucleotides with a frequency of 1 in 300 [16], which would result in >500 mismatches per genome, HSV-1 may have adapted cellular MMR to prevent the maintenance of mutations generated during DNA replication.

Cellular MMR has been closely linked to DNA replication [38–40] and the cellular sliding clamp PCNA recruits MMR proteins to replicating cellular DNA [41]. Consistent with the potential involvement of PCNA in MMR of viral DNA, we observe high levels of PCNA and the clamp loader complex (RFC1-5) at replication forks (Fig 2) and observe similar pulse chase kinetics (Fig 3B) and localization with sites of DNA synthesis (Fig 4) for PCNA as compared to MMR proteins. Furthermore, PCNA association with viral DNA depends on active viral DNA synthesis (S3 Fig and S4 Fig) and PCNA knockdown results in reduced virus yield [42]. Taken together, these data strongly implicate cellular PCNA and MMR in the maintenance of viral genome integrity during viral DNA replication (Fig 6B).

### Double Strand Break Repair

The HSV-1 genome contains several nicks and gaps [17–19], which likely cause DSBs during DNA replication. Another source of DSBs on viral DNA is through the actions of type II topoisomerases, which cause transient DSBs to relax supercoiling that occurs during transcription and replication or to allow for the decatenation of linked DNA molecules [43]. In support of this, type II topoisomerases TOP2A and TOP2B are associated with viral replication forks (Fig 2). DSBs are required for HSV-1 genome isomerization [44] but would likely impede transcription and replication of viral DNA.

Here we identified MRE11 and RAD50 components of the MRN (MRE11-RAD50-NBS1) complex at viral replication forks (Fig 2). Furthermore, XRCC5 and 6 were identified just below the enrichment threshold (S1 Table). These proteins play a role in processing DSBs before repair. Although HSV-1 inhibits cellular DSB repair by nonhomologous end joining and homologous recombination [1], alternative modes of recombination have been suggested through the actions of the viral alkaline nuclease (UL12) and ICP8 [45]. UL12 was not identified to be associated with viral replication forks in our assays but has previously been shown to coprecipitate with ICP8 [36]. MRN complex members colocalize with ICP8 in virus infected cells throughout infection and infections in MRE11 defective cells result in reduced viral yields [46,47]. Therefore, this complex likely plays a role in repair or stabilization of DSBs on viral DNA during replication or may act upstream of virus specific recombination events (Fig 6B).

### Single Strand Break Repair

SSBs can form on viral DNA during lagging strand synthesis or by the actions of topoisomerase I (TOP1), which was enriched on viral replication forks (Fig 2). TOP1 cleaves a single strand of DNA to create a transient break to allow for topological changes during replication or transcription. During the repair of TOP1 associated SSBs, PARP1 and RECQL inhibit replication restart, DNA ends are repaired by PNKP and the nick is ligated through the actions of XRCC1 and LIG3 [43]. In this study, all of these factors were enriched on viral replication forks (Fig 2). Furthermore, pulse chase kinetics suggest that some of these factors are most closely associated with sites of active DNA synthesis (Fig 3B). PARP1, XRCC1, and LIG3 are also involved in the repair of nicks left behind after base excision repair, which is inhibited in our assays. It is therefore likely that SSB repair of TOP1 intermediates occurs near viral replication forks (Fig 6B). Some of these proteins may also play a role in the repair nicks left behind during lagging strand synthesis of Okazaki fragments.

### Chromatin Remodeling Factors May Regulate Repair or Transcription of Nascent DNA

INO80 (INO80, RUVBL2, RUVBL1), NuRD (HDAC1, HDAC2, RBBP4, CHD3, CHD4, MTA1, MTA2), and FACT (SSRP1, SUPT16H) chromatin remodeling complex members were selectively enriched on viral replication forks (Fig 2 and S2A and S2B Fig). The NuRD and INO80 complexes catalyzes ATP-dependent chromatin remodeling and play roles in cellular transcription repression and activation, DNA replication, recombination, and repair [48–50]. The FACT complex remodels nucleosomes and tethers histones to prevent their removal during transcription elongation [51]. Although histones in the form of nucleosomes do not appear to be associated with replicated viral DNA, histone H1 and H2A variants are associated with newly replicated viral DNA (S2A and S2B Fig). The functions of chromatin remodeling complexes and histones in the regulation of processes that occur on viral DNA, including replication-coupled events, is of great interest for future studies.

### Transcription Factor Interactions Provide Insight into Mechanisms that Couple Viral DNA Replication and Late Gene Transcription

Many DNA viruses undergo replication-coupled late gene expression, although the mechanisms that either repress transcription before or activate transcription after replication are not well understood. Interestingly, we identified cellular transcription factors associated with viral replication forks including Mediator, TFIID, Pol II, ICP4, Integrator, SUPT5H, and SUPT6H (Fig 2). These factors have known functions in transcription initiation and elongation of cellular genes. The promoters of late genes are relatively simple and contain only initiator and TATA containing elements [52,53] and lack sites for Sp1, which are found within promoters of IE and early genes. We did not identify Sp1 in our assays, suggesting that proteins associated with late and not IE or early gene promoters are enriched in our assays. Late gene promoters are bound by ICP4, TBP, and Pol II prior to the onset of DNA replication [54], suggesting a model by which preinitiation complexes (PICs) begin to form before DNA replication. It is therefore likely that genome replication enhances recruitment and some concurrent event promotes promoter conformations that are conducive to initiation and promoter escape (Fig 6C).

The dynamics of transcriptional events with respect to replication forks suggests the involvement of the Mediator complex in the regulation of late gene transcription. In this study, we reveal a strong association of Mediator with viral replication forks (Fig 2). Pulse chase studies reveal a preference for binding at replication forks, with Mediator levels decreasing in chases (Fig 3D). This observation is especially robust for the MED12 and MED13 subunits of the complex. MED12 and MED13 are components of the kinase domain, which sterically blocks interactions with between Mediator and the C-terminal domain (CTD) of Pol II to repress initiation and reinitiation events [55]. Mediator compositional changes have recently been shown to occur at the promoters of cellular genes, which potentially coordinate sequential events in transcription activation [56]. Mediator containing the kinase module first associates with activators bound to enhancers, upon PIC formation the kinase domain leaves allowing Mediator to associate with the CTD of Pol II, which is followed by CTD phosphorylation and promoter escape. Taken together, we propose a testable model whereby Mediator containing MED12 and MED13 associates near the promoters of late viral genes during DNA replication and that conformational changes that occur within Mediator promote transcriptional activation (Fig 6C).

It is likely that ICP4 is involved in the recruitment of Mediator to replication forks. ICP4 interacts with Mediator and is required for the recruitment of Mediator to the promoters of all classes of viral genes [57–59]. Furthermore, the form of Mediator that associates with ICP4 contains MED12 and MED13 and lacks MED26, which is consistent with the form we found associated with viral replication forks.

Mediator was more enriched on genomes pulse labeled for 20 min at 4 hpi as compared to genomes labeled from 4–6 hpi (Fig 3C), suggesting that initial rounds of replication are sufficient to drive conformation changes within the promoters of late viral genes. To test this hypothesis, we carried out RNA-seq to assay for late viral gene expression as a function of time of inhibition of viral DNA replication (Fig 5). Our data indicate that inhibition of replication after initial rounds (at 3 or 4 hpi) results in a significant reduction of the number of viral genomes present at 12 hpi (A), but has little effect on the number of late gene transcripts that are expressed at that time (B,D,E). These data indicate that initial rounds of viral DNA replication are sufficient to drive robust late gene expression, further supporting a role of replication in promoting conformational changes within promoters of late viral genes.

Taken together, our results provide novel insight into the coupling of viral DNA replication with several fundamental processes that occur on the HSV-1 genome during productive infection. Imaging studies reveal functional subdomains within viral replication compartments where DNA synthesis and factors that participate in replication-coupled events are concentrated (Fig 6A). Results also support models whereby dynamic associations of cellular factors with viral replication forks coordinate viral DNA replication with the repair and maintenance of viral DNA (B) and the expression of late viral genes (C).

## Materials And Methods

### Cells and Viruses

Experiments were performed using MRC5 (human fetal lung) or Vero (African green monkey kidney) cells obtained from and propagated as recommended by ATCC. The viruses used in this study include the HSV-1 wild type strain KOS and UL2/UL50 [26]. EdC-prelabeled virus was prepared as described previously [26].

### Quantification of Viral DNA

To measure DNA quantity for the calculation of viral replication rates (Fig 1A), 5x10^5^ Vero or MRC5 cells were infected with KOS or UL2/UL50 at a multiplicity of infection (MOI) of 10 plaque forming units (PFU)/cell. DNA was collected at 0, 2, 4, 6, 8, 10, 12, and 24 hpi by aspirating growth medium and collecting infected cells in 0.2 ml DNA extraction buffer (0.5% SDS, 400 μg/ml proteinase K, 100 mM NaCl). Samples were incubated at 37°C for 4 hours followed by heat inactivation at 65°C for 15 min and phenol:chloroform extraction. DNA was diluted 1:500 in water and qPCR was carried out to determine the number of viral genomes using primers specific for the HSV-1 tk gene [31]. Standard curves were generated using purified KOS DNA. The rates of viral DNA replication were calculated as bp synthesized per min using the following formula: $\frac{152,000\;bp/genome}{\frac{duration}{\log2\left(final\;number\;of\;genomes\right)-log2(initial\;number\;genomes)}}$

To measure genome quantity for comparison with RNA-Seq data (Fig 5A), 2x10^6^ Vero cells were infected with KOS at an MOI of 10 PFU/cell and incubated at 37°C for 12 hours in the presence or absence of 100 μM acyclovir. DNA extraction and qPCR were carried out as above using primers specific for the HSV-1 gC gene [31].

### Isolation of Viral Replication Forks and Associated Proteins

Viral DNA isolation was carried out using the aniPOND technique as described previously [27] with the following modifications. A confluent monolayer of ~7x10^7^ MRC5 cells was infected with the UL2/UL50 virus at an MOI of 10 PFU/cell for one hour at room temperature. After adsorption, the inoculum was removed and cells were rinsed with room temperature tris-buffered saline (TBS) before growth medium was replaced. Cells were incubated at 37°C for four hours before adding 25 μM EdC (Sigma-Aldrich) for 5, 20, or 120 min. Chases were carried out after a 20 min pulse by quickly rinsing cells three times with chase medium containing 150 μM 2´deoxycytidine (deoxyC) followed by incubation in chase medium for an additional 40 min. Negative control samples were not labeled with EdC. Harvesting nuclei, cell washes, and biotin conjugation by click chemistry were carried out as described. For cell lysis and DNA fragmentation, resuspended cell pellets were incubated for 45 min in lysis buffer and sonicated 6 times for 30 sec each at 40% amplitude using a Sonics Vibra Cell Ultra Sonic Processer equipped with a 3 mm microtip probe. DNA fragments ranged in size from 100–500 bp. Dynabeads MyOne Streptavidin T1 (ThermoFisher) were used to purify biotinylated DNA-protein complexes. Proteins were eluted from streptavidin-coated beads by boiling in 2x SDS Laemmli sample buffer. DNA isolation from input and bound samples was carried out as described [26]. Experiments yielded ~6 μg total DNA and 100–200 ng bead bound DNA.

### Mass Spectrometry and Data Analysis

Mass spectrometry was carried out by MSBioworks as described [26]. Proteins were considered enriched by aniPOND based on the following criteria: 1) protein had at least 5 SpCs in one experimental condition per data set, 2) protein was not detected in the negative control or was enriched over the negative control by at least four-fold based on dividing SpC values, and 3) protein was detected in duplicate experiments. The normalized spectral abundance factor (NSAF: $\frac{SpC/MW}{\Sigma (SpC/MW)}$) was calculated for each sample to account for differences in protein size and total protein yield. Average NSAF from two independent data sets were used for all analyses. Raw and normalized SpC data for enriched proteins are provided in S1 Table.

### Imaging of Viral DNA and Immunofluorescence

Click chemistry and immunofluorescence experiments were carried out after infection of Vero cells with KOS or EdC-labeled KOS as described [26]. To pulse label viral replication forks, 25 μM EdC was added to KOS infected cells at 4 hpi for 20 min before fixation with 3.7% paraformaldehyde. The Click-iT Alexa Fluor 488 Imaging Kit (ThermoFisher) was used to tag EdC labeled viral genomes and immunofluorescence was carried out using the following primary antibodies and dilutions: mouse anti-ICP8: ab20194 (Abcam), 1:200; mouse anti-PCNA: sc-056 (Santa Cruz), 1:200; mouse anti-MLH1: 550838 (BD Biosciences), 1:200; mouse anti-MSH2: Ab-2 NA27 (Calbiochem), 1:100; rabbit anti-MSH6: A300-023A (Bethyl Laboratories), 1:100; mouse anti-UL42: 2H4 ab19311 (Abcam), 1:200; mouse anti-ICP4: 58S, 1:500; mouse anti-Pol II (POLR2A): 4H8 (Abcam), 1:500; mouse anti-Sur2 (MED23): 550429 (BD Biosciences), 1:500; rabbit anti-Trap220 (MED1): sc-8998 (Santa Cruz), 1:250; rabbit anti-Spt5 (SUPT5H): A300-869A (Bethyl Laboratories), 1:200; and rabbit anti-Spt6 (SUPT6H): ab32820 (Abcam), 1:200. Goat anti-mouse and anti-rabbit alexa fluor 594-conjugated secondary antibodies (Santa Cruz) were diluted 1:500.

### RNA Isolation and RNA-Seq

Monolayers of 2x10^6^ Vero cells were infected with KOS at an MOI 10 PFU/cell and incubated at 37°C for 12 hours. Acyclovir (100 μM) was added at the indicated times post infection. RNA was isolated using the Ambion RNAqueous-4PCR Kit and quantified using the Agilent RNA 6000 Nano Kit. RNA-Seq was carried out as described [31] and sent to the Tufts University Core Facility for sequencing. Illumina reads were processed and aligned to the KOS genome using CLC Workbench V8. To account for variations in sample reads, each sample was normalized to the total number of reads.

## Supporting Information

S1 Table Raw SpC and NSAF Data Used for Proteomic Analyses. (XLSX)

S1 Fig Cellular DNA is Not Efficiently Labeled with EdC during Pulse Chase Experiments. (PDF)

S2 Fig Viral Genome Dynamics during Pulse Chase Experiments. (PDF)

S3 Fig Analysis of the Colocalization of Viral Replication Fork Associated Proteins with Pre-Replicative Viral Genomes at 2 hpi. (PDF)

S4 Fig Analysis of the Colocalization of Viral Replication Fork Associated Proteins with Viral Genomes Blocked for DNA Synthesis. (PDF)
