# Supplementary material for: Replication-Coupled Recruitment of Viral and Cellular Factors to Herpes Simplex Virus Type 1 Replication Forks for the Maintenance and Expression of Viral Genomes
Source: PLoS Pathog. 2017 Jan 17;13(1):e1006166. doi: 10.1371/journal.ppat.1006166 (PMC5271410; doi:10.1371/journal.ppat.1006166)
Supplement: S1 Fig — (PDF) [file ppat.1006166.s002.pdf]

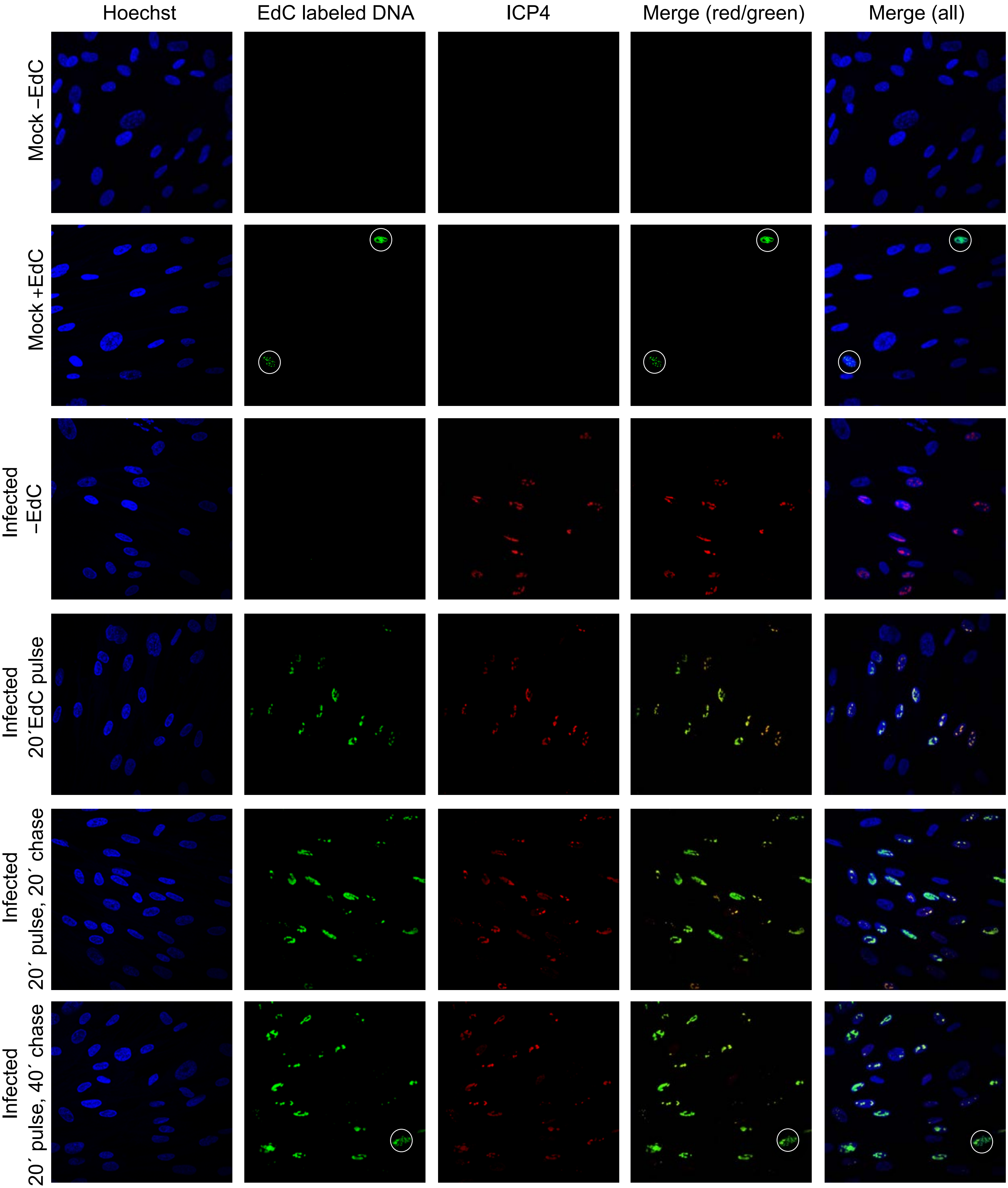

Hoechst
EdC labeled DNA
ICP4
Merge (red/green)
Merge (all)
Mock –EdC
Mock +EdC
Infected –EdC
Infected 20´EdC pulse
Infected 20´ pulse, 20´ chase
Infected 20´ pulse, 40´ chase

S1 Figure. Cellular DNA is Not Efficiently Labeled with EdC during Pulse Chase Experiments. MRC5 cells were grown to confluency and infections were carried out using conditions that mimic viral replication fork pulse chase experiments for isolation of viral DNA and associated proteins. Cells were mock infected or infected with UL2/UL50 at an MOI of 10 PFU/cell. After four hours, cells were pulse labeled with (+) or without (-) EdC for 20 min (20´ pulse) and either fixed or chased with deoxyC for 20 (20´ chase) or 40 (40´ chase) min followed by fixation, covalent tagging of labeled DNA with alexa fluor 488, and immunofluorescence. EdC-labeled viral DNA (green) colocalizes with ICP4 (red) and EdC-labeled cellular DNA (green, circled) colocalizes with Hoechst stain (blue). ~100 cell were counted to determine the percentage of cells with labeled cellular DNA. Less than 13% of mock infected cells incorporated EdC into cellular DNA and less than 2% of infected cells incorporated EdC into cellular DNA. This is because HSV-1 infection inhibits cellular DNA synthesis. All viral replication compartments (marked by ICP4 staining) were labeled with EdC.
