# Supplementary material for: Replication-Coupled Recruitment of Viral and Cellular Factors to Herpes Simplex Virus Type 1 Replication Forks for the Maintenance and Expression of Viral Genomes
Source: PLoS Pathog. 2017 Jan 17;13(1):e1006166. doi: 10.1371/journal.ppat.1006166 (PMC5271410; doi:10.1371/journal.ppat.1006166)
Supplement: S2 Fig — (PDF) [file ppat.1006166.s003.pdf]

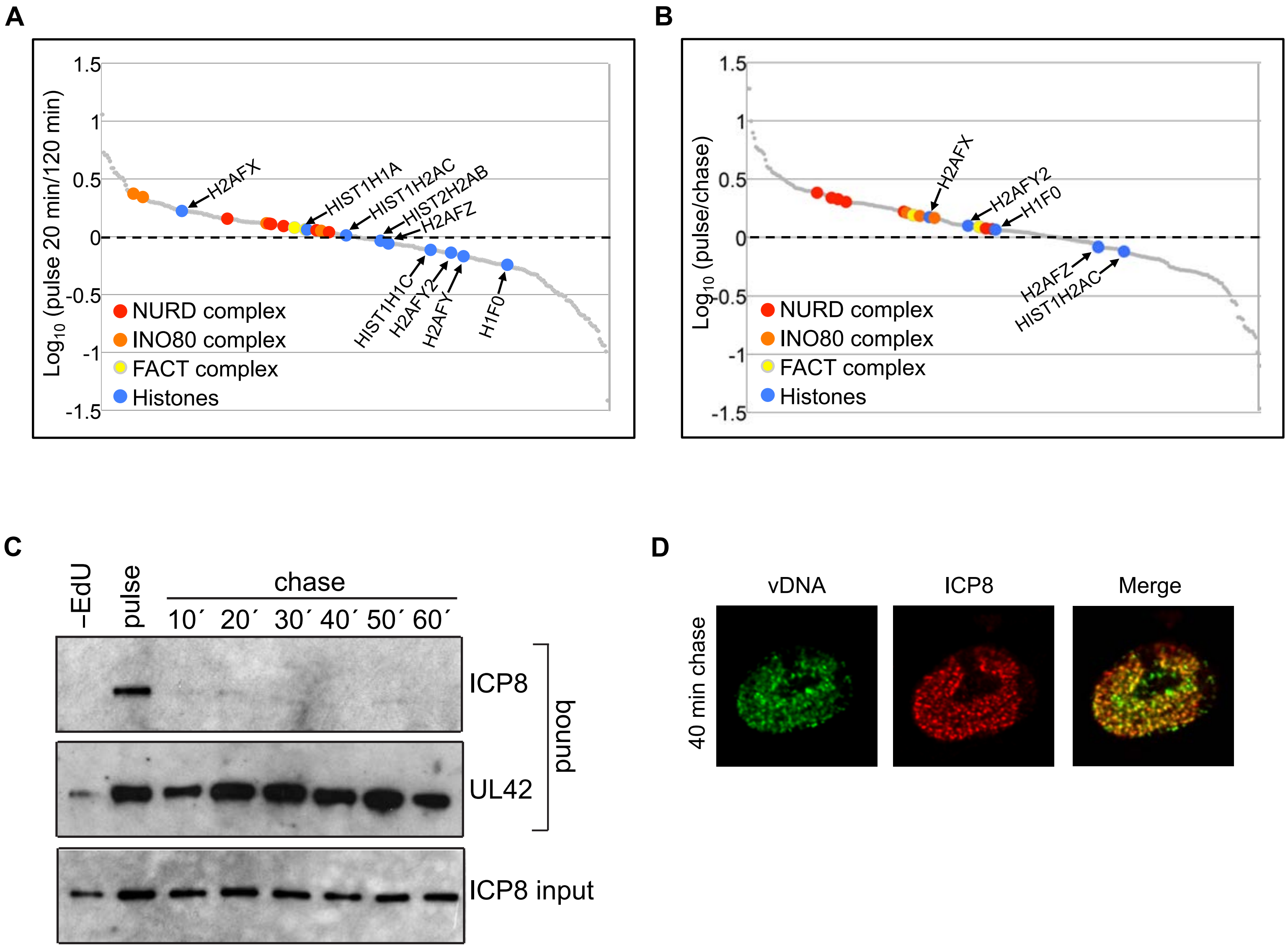

S2 Figure. Viral Genome Dynamics during Pulse Chase Experiments. A. Pulse and B. pulse-chase analysis of viral replication forks reveals interaction dynamics between cellular chromatin remodeling factors and histones and nascent viral DNA. C. Western blot of replication fork associated proteins. Viral replication forks were pulse labeled with EdU for 10 min (pulse) and chased for 0-60 min (chase). Labeled DNA was tagged with biotin and DNA-protein complexes were purified as described in the Materials and Methods. Western blotting was carried out on cell lysates (ICP8 input) to compare total protein yield and on proteins eluted from purified DNA (ICP8 and UL42 bound). For the negative control, assays were carried out in the absence of EdU (-EdU). D. Chasing the fate of nascent viral DNA. Viral DNA (vDNA) was pulse labeled for 20 min with EdC as in Figure 4 followed by a 40 min chase with deoxyC. Cells were fixed followed by alexa fluor tagging of EdC-labeled viral DNA (green) and immunofluorescence for ICP8 (red) as described in the Materials and Methods.
