# Supplementary material for: Replication-Coupled Recruitment of Viral and Cellular Factors to Herpes Simplex Virus Type 1 Replication Forks for the Maintenance and Expression of Viral Genomes
Source: PLoS Pathog. 2017 Jan 17;13(1):e1006166. doi: 10.1371/journal.ppat.1006166 (PMC5271410; doi:10.1371/journal.ppat.1006166)
Supplement: S3 Fig — (PDF) [file ppat.1006166.s004.pdf]

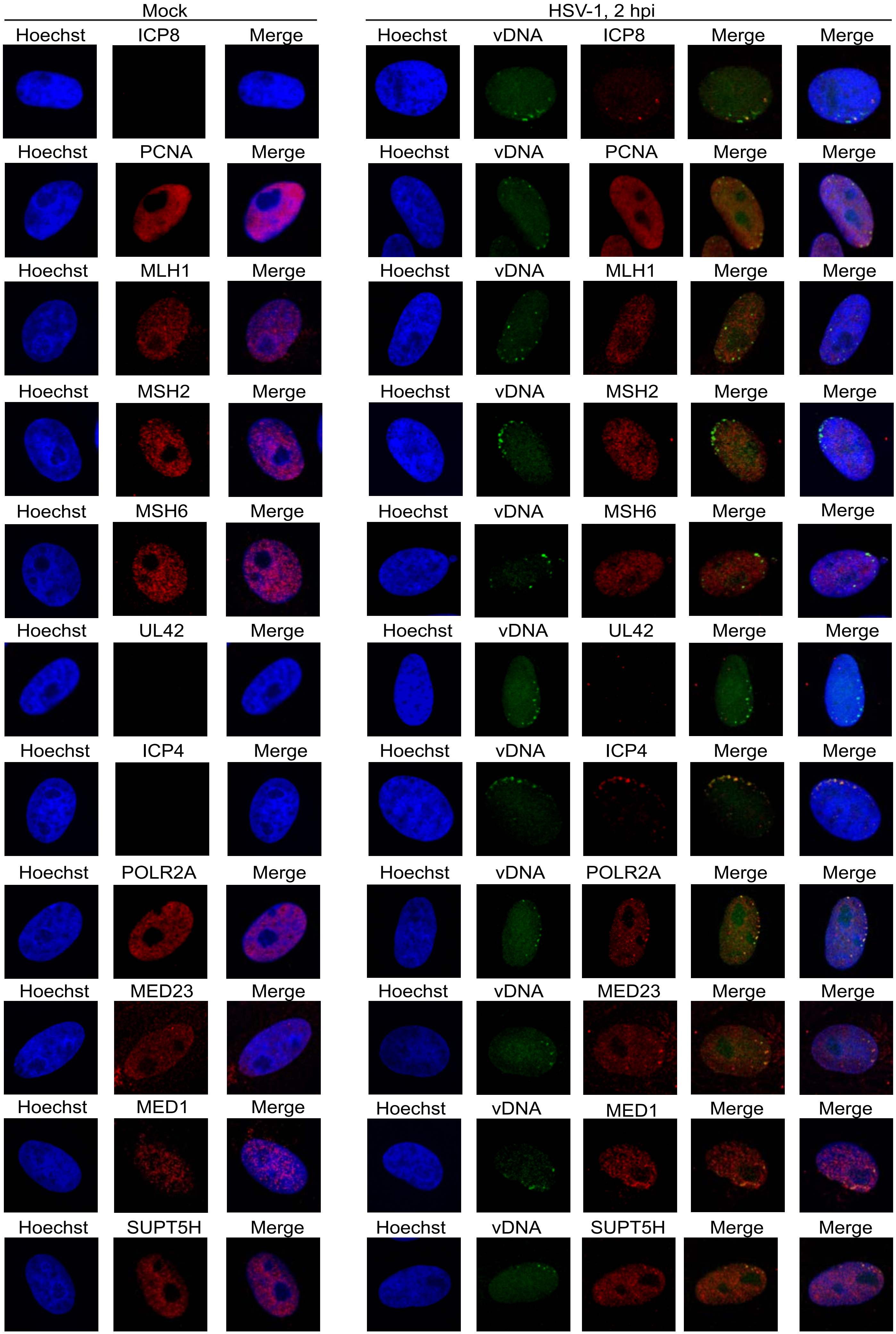
Mock
HSV-1, 2 hpi
Hoechst
ICP8
Merge
vDNA
PCNA
MLH1
MSH2
MSH6
UL42
ICP4
POLR2A
MED23
MED1
SUPT5H

S3 Figure. Analysis of the Colocalization of Viral Replication Fork Associated Proteins with Pre-Replicative Viral Genomes at 2 hpi. Vero cells were either mock infected or infected with EdC labeled KOS at an MOI of 10 PFU/cell. After two hours, cells were fixed and EdC labeled DNA was tagged with alexa fluor 488 to visualize pre-replicative viral genomes (green). Viral and cellular proteins were visualized by immunofluorescence (red). Nuclei were labeled with Hoechst (blue). Gene names that correspond to detected proteins are indicated for consistency with proteomics data. See also Table 1.
