# Supplementary material for: Replication-Coupled Recruitment of Viral and Cellular Factors to Herpes Simplex Virus Type 1 Replication Forks for the Maintenance and Expression of Viral Genomes
Source: PLoS Pathog. 2017 Jan 17;13(1):e1006166. doi: 10.1371/journal.ppat.1006166 (PMC5271410; doi:10.1371/journal.ppat.1006166)
Supplement: S4 Fig — (PDF) [file ppat.1006166.s005.pdf]

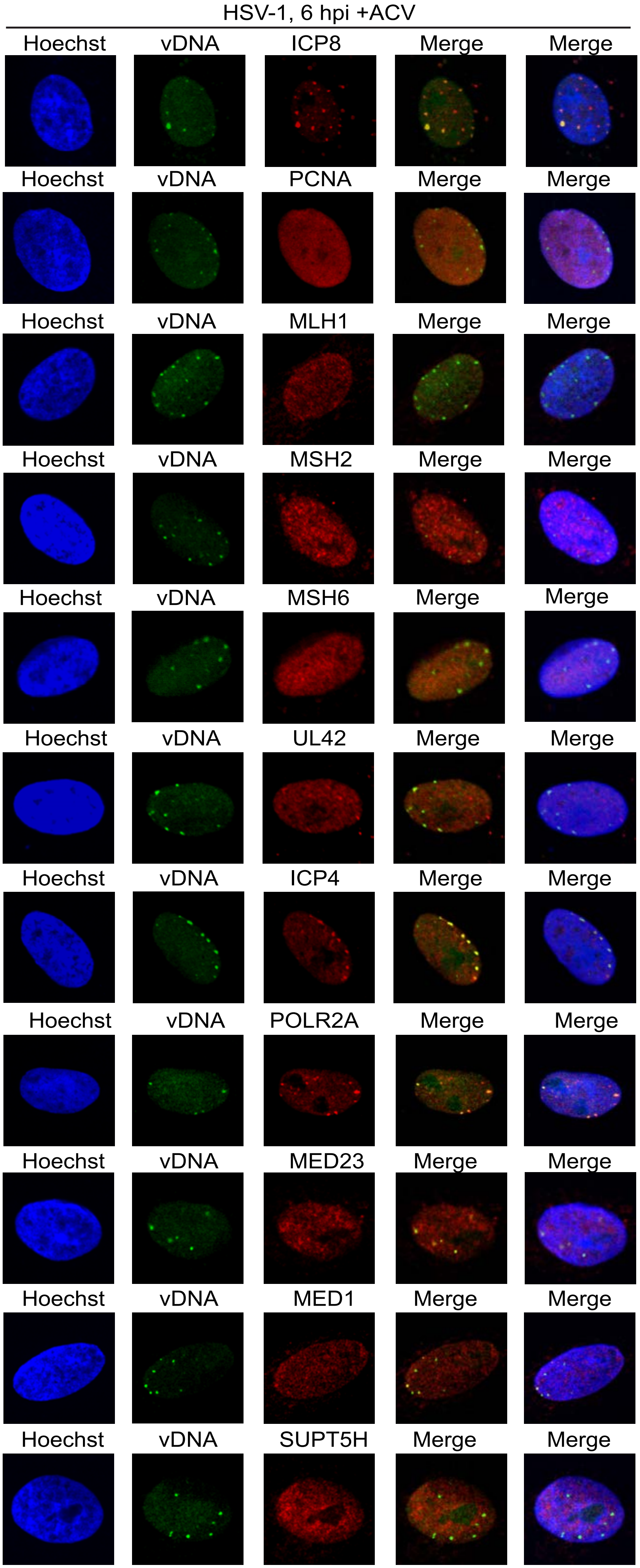
HSV-1, 6 hpi +ACV
Hoechst
vDNA
ICP8
Merge
Merge
PCNA
MLH1
MSH2
MSH6
UL42
ICP4
POLR2A
MED23
MED1
SUPT5H

S4 Figure. Analysis of the Colocalization of Viral Replication Fork Associated Proteins with Viral Genomes Blocked for DNA Synthesis. Vero cells were infected with EdC labeled KOS at an MOI of 10 PFU/cell in the presence of 100 μM acyclovir (ACV). After six hours, infected cells were fixed and EdC labeled DNA was tagged with alexa fluor 488 to visualize viral genomes (green). Viral and cellular proteins were visualized by immunofluorescence (red). Nuclei were labeled with Hoechst (blue). Gene names that correspond to detected proteins are indicated for consistency with proteomics data. See also Table 1.
